# Pertussis hospitalizations among term and preterm infants: clinical course and vaccine effectiveness

**DOI:** 10.1186/s12879-019-4563-5

**Published:** 2019-10-29

**Authors:** Nicoline A. T. van der Maas, Elisabeth A. M. Sanders, Florens G. A. Versteegh, Albertine Baauw, Anneke Westerhof, Hester E. de Melker

**Affiliations:** 1 Centre for Infectious Disease Control, National Institute for Public Health and the Environment, PObox 1, 3720BA, Bilthoven, The Netherlands; 2 Department of Paediatric Immunology and Infectious Diseases, University Medical Hospital Utrecht, Utrecht, the Netherlands; 3 University of Groningen, University Medical Centre Groningen/Beatrix Children’s Hospital, Groningen, the Netherlands; 4 Department of Paediatrics, Rijnstate Hospital, Arnhem, the Netherlands

**Keywords:** Pertussis, Preterms, Infants, Hospitalization, Vaccine effectiveness, Vaccination

## Abstract

**Background:**

Pertussis causes severe disease in young unvaccinated infants, with preterms potentially at highest risk. We studied pertussis in hospitalized infants as related to gestational age (GA) and vaccination history.

**Methods:**

Medical record data of 0-2y old patients hospitalized for pertussis during 2005–2014 were linked to vaccination data. Multivariable logistic regression was used to study the association between GA and vaccination history on the clinical disease course. We compared vaccine effectiveness (VE) against hospitalization for pertussis between term and preterm infants (i.e., <37w GA) using the screening method as developed by Farrington.

**Results:**

Of 1187 records, medical data from 676 were retrieved. Of these, 12% concerned preterms, whereas they are 8% of Dutch birth cohorts. Median age at admission was 3 m for preterms and 2 m for terms (*p* < 0.001). Preterms more often had received pertussis vaccination (62% vs 44%; *p* = 0.01) and more often had coinfections (37% vs 21%; p = 0.01). Preterms tended more often to have complications, to require artificial respiration or to need admittance to the intensive care unit (ICU). Preterms had longer ICU stays (15d vs 9d; *p* = 0.004).

Vaccinated preterms and terms had a lower median length of hospital stay and lower crude risks of apneas and the need for artificial respiration, additional oxygen, and ICU admittance than those not vaccinated. After adjustment for presence of coinfections and age at admittance, these differences were not significant, except the lower need of oxygen treatment in vaccinated terms. Effectiveness of the first vaccination against pertussis hospitalizations was 95% (95% CI 93–96%) and 73% (95% CI 20–91%) in terms and preterms, respectively. Effectiveness of the second dose of the primary vaccination series was comparable in both groups (86 and 99%, respectively).

**Conclusions:**

Infants hospitalized for pertussis suffer from severe disease. Preterms were overrepresented, with higher need for intensive treatment and less VE of first vaccination. These findings stress the need for alternative prevention, in particular prenatal vaccination of mothers, to reduce pertussis in both groups.

## Background

Pertussis is a highly contagious respiratory tract infection, caused mainly by *Bordetella pertussis* and less frequently by *Bordetella parapertussis* [1]. In the pre-vaccination era, infants and children contracted pertussis in their first years of life, with a clinical course characterized by uncontrollable coughing attacks, often accompanied by paroxysms, post-tussive vomiting, and inspiratory whooping. Consistently high vaccination coverage has substantially decreased pertussis in the population [2, 3], but newborns too young to be vaccinated remain at high risk for severe complications including apnea, cyanosis, pneumonia, encephalopathy or even death [4]. This risk is increasing due to the worldwide pertussis reemergence in the 1990s, even in areas of high vaccination coverage in all age groups, with transmission of disease from household members to newborns. Today, high pertussis incidences in infants are observed, with incidence peaking every two to three years [3, 5, 6]. Worldwide in 2014, an estimated 24 million cases and 160,000 deaths from pertussis occurred in children younger than 5 years, with the African region contributing the greatest share [7]. In the Netherlands, each year approximately 150–180 children <2y are hospitalized and one infant, in general too young to be vaccinated, dies due to pertussis [8]. For this reason, many countries are discussing prenatal pertussis vaccination of mothers to protect newborns, and a growing number of countries now recommend it [9]. This measure is effective in preventing pertussis in the first months of life and has decreased the pertussis disease burden in young infants [10, 11]. In the Netherlands, the Health Council advised that 3rd trimester maternal pertussis vaccination be offered. This is overall very effective in prevention of pertussis in early infancy, but preterms may benefit less due to a smaller time-window for mother-to-child transfer of antibodies before delivery [12, 13]. However, vaccine effectiveness (VE) is reportedly lower after 2nd trimester pertussis vaccination [14]. Given the introduction of a maternal vaccination strategy against pertussis in The Netherlands, we sought to gain more insight into the current pertussis burden among hospitalized infants, with special attention to preterms.

## Methods

### Setting, data collection, and linkage

During the study period (2005–2014), the Netherlands’ National Immunization Programme included a 3 + 1 infant vaccination schedule using pentavalent (2005–2011) or hexavalent (2012–2014) combination vaccines containing acellular pertussis, with doses at 2, 3, 4 and 11 months of age [15]. Vaccination coverage of the infant series was 93.5–95.5% for all included birth cohorts [2].

We sent a letter with information about the study aim and logistics together with an informed consent form to the boards of all hospitals in the Netherlands. To those that supplied written approval, we sent a list of all records selected from the National Registry of Hospital Care. The relevant medical records were located and data extracted by trained medical students, supervised by a medical doctor (NvdM). Besides birth date, sex, and postal code, data were collected on gestational age (GA) and birth weight, clinical symptoms at admission, date of admission and discharge, diagnostics, and details about the medical situation, complications, treatments, and clinical status at discharge.

In the National Registry of Hospital Care and the vaccination registry, which includes all 0–18-year-olds and any changes in residence, pseudonyms were created based on birth date, sex, and postal code. For infants who moved over time, pseudonyms in the vaccination registry reflected their known postal codes to a maximum of six*.* Using the pseudonyms, medical record data were linked to the national vaccination registry.

To ensure privacy, a Trusted Third Party was used for certain steps in data collection and data linking. Researchers were allowed to use age only in months.

Medical ethical approval was not needed because no one was subjected to imposed rules or acts. According to Dutch law, informed consent of patients was not required because the study served public interest, and asking permission was not feasible [16, 17].

### Data sources

#### National Registry of hospital care

Hospital Care data include the main diagnosis, date of birth, four digits of the postal code, sex, and date of admission or outpatient treatment [18]. We located medical records for 0–2-year-olds with a primary diagnosis of whooping cough between 2005 and 2014 based on the International Classification of Diseases (ICD) codes, i.e., ICD-9 0330 or ICD-10 A370 (caused by *B. pertussis*); ICD-9 0331 or ICD-10 A371 (*B. parapertussis*); ICD-9 0338 or ICD-10 A378 (other specified organism); or ICD-9 0339 or ICD-10 A379 (other unspecified organism).

### Statistical analysis

Differences in general characteristics and clinical aspects of pertussis between terms and preterms (defined as born before 37w GA) were described and tested using Pearson’s Chi Square or Fisher’s exact test for dichotomous and categorical variables, and student t-test or Wilcoxon rank test for continuous variables.

Multivariable logistic regression was performed to assess the association between prematurity and the clinical picture of pertussis and to study the association between pertussis vaccination and clinical characteristics stratified by GA. Data for both analyses were adjusted for age at hospitalization and coinfections.

Vaccine effectiveness (VE), stratified for preterms and terms, was computed using the screening method as developed by Farrington [19]. We used monthly cumulative coverage estimates of a timely first dose, stratified by preterms and terms [20]. In the main analysis, we classified children without exact GA but with written information of a term pregnancy and infants without information on GA as terms. In sensitivity analyses, we included only children with known GA.

Analyses were performed using SAS version 9.4. A *p*-value < 0.05 was considered statistically significant. Furthermore, term infants were set as reference in all analyses.

## Results

### General descriptives

We invited the participation of 87 hospitals. Of these, 4/8 university hospitals, 19/26 top clinical hospitals, and 27/51 local hospitals participated. Overall, data of 57% of eligible cases (676/1187) were available.

Of the 676 hospitalized pertussis cases, 80 infants (12%; 95%CI 10–14%) were born preterm, 388 (57%) were born term, and 208 (31%) lacked information on gestational age (GA) at birth.

Median GA of preterms at birth was 35w (range 26w – 36w). Thirteen (16%) preterms were born before 32w GA, whereas 67 (84%) were born between 32w – 36w GA.

Among terms and preterms, respectively, 81% and 75% of medical records could be linked to vaccination data. Pseudonyms of the remaining records were too unspecific for reliable linkage.

### Main analyses (*n* = 676)

#### Hospitalization

Median age at hospitalization was 2 months (Table 1). Terms were hospitalized at younger ages than preterms (median 2.0 m vs 3.0 m). Median duration of hospitalization was 5 and 6 days for terms and preterms, respectively. Overall, 46% of infants were vaccinated at admission, with statistically significant higher frequencies in preterms (62%) than in terms (44%; *p* = 0.01). Among vaccinated infants, the median interval between first vaccination and hospitalization was 35d in terms (mean 106d), compared with 37d in preterms (mean 105d).

**Table 1:** Clinical course, diagnostic tests, and treatment of pertussis in term and preterm infants. Denominator is the number of infants in the group, which is noted in column headings or the respective cells

	Total group (*n* = 676)	Probable and certain term infants (*n* = 596)^a^	Certain term infants (*n* = 388)^b^	Preterm infants (*n* = 80)
Characteristics of hospitalization, clinical course, and treatment				
Boys; *n* (%)^1^	335 (49.6%)	295 (49.5%)	185 (47.7%)	40 (50%)
Age in months at admittance; median (range)	2.0 (0–36)	2.0 (0–35)	2.0 (0–35)	3.0 (0–25)^c,d^
Duration of hospitalization in days; median (range)	5.0 (1–51)	5.0 (1–51)	5.0 (1–51)	6.0 (1–49)
Admission intensive care unit (ICU); *n* (%)	69 (10.2%)	59 (9.9%)	50 (12.9%)	10 (12.5%)
Duration ICU stay in days; median (range)	9 (2–34)	9 (2–25)	9 (2–25)	15 (8–34)^c,d^
Vaccinated at admission; *n* (%)	250/541 (46.2%)	213/481 (44.3%)	130/319 (40.7%)	37/60 (61.7%)^c,d^
Coughing attacks; *n* (%)	494 (73.1%)	438 (73.5%)	280 (72.2%)	56 (70.0%)
Apnea; *n* (%)	110 (16.3%)	92 (15.4%)	71 (18.3%)	18 (22.5%)
Whooping; *n* (%)	24 (3.6%)	23 (3.9%)	18 (4.6%)	1 (1.3%)
Vomiting; *n* (%)	238 (35.2%)	210 (35.2%)	146 (37.6%)	28 (35.0%)
Prolonged inspiratory effort; *n* (%)	49 (7.3%)	42 (7.1%)	33 (8.5%)	7 (8.8%)
Collapse; *n* (%)	8 (1.2%)	6 (1.0%)	5 (1.3%)	2 (2.5%)
Cyanosis; *n* (%)	284 (42.0%)	259 (43.5%)	176 (45.4%)	25 (31.3%)^c,d^
Fever; *n* (%)	60 (8.9%)	52 (8.7%)	33 (8.5%)	8 (10.0%)
Feeding problems; *n* (%)	212 (31.4%)	185 (31.0%)	133 (34.3%)	27 (33.8%)
Any complication^e^; *n* (%)	63 (9.3%)	53 (8.9%)	39 (10.1%)	10 (12.5%)
Antibiotics before admission^f^; *n* (%)	131/657 (19.9%)	114/580 (19.7%)	66/377 (17.5%)	18/77 (23.4%)
Antibiotics during admission^g^; *n* (%)	543/674 (80.5%)	475/587 (80.9%)	313/382 (81.9%)	68/78 (87.2%)
Intravenous antibiotics during admission; *n* (%)	12/403 (3.0%)	9/357 (2.5%)	6/244 (2.5%)	3/46 (6.5%)
Antibiotic use in days; median (range)	6 (0–28)	6 (0–28)	5 (0–28)	5.5 (1–18)
Artificial respiration; *n* (%)	50/654 (7.7%)	39/577 (6.8%)	32/375 (8.5%)	11/77 (14.3%)^c^
Additional oxygen; *n* (%)	226/661 (34.2%)	198/585 (33.9%)	150/382 (39.3%)	28 (36.8%)
Symptoms remaining; *n* (%)	518 (76.6%)	460 (77.2%)	298 (76.8%)	58 (72.5%)
Re-admittance <6w after discharge; *n* (%)	94 (14.0%)	85 (14.3%)	56 (14.4%)	9 (11.4%)
Diagnostics				
Pertussis test: culture, PCR and serology; *n* (%)	34 (5.0%)	31 (5.2%)	24 (6.2%)	3 (3.8%)
Pertussis test: culture and PCR; *n* (%)	59 (8.7%)	52 (8.7%)	40 (10.3%)	7 (8.8%)
Pertussis test: PCR and serology; *n* (%)	84 (12.4%)	73 (12.2%)	41 (10.6%)	11 (13.8%)
Pertussis test: culture and serology; *n* (%)	24 (3.6%)	24 (4.0%)	17 (4.4%)	0 (0%)
Pertussis test: culture; *n* (%)	37 (5.5%)	29 (4.9%)	18 (4.6%)	8 (10%)
Pertussis test: PCR; *n* (%)	329 (48.7%)	290 (48.7%)	194 (50%)	39 (48.8%)
Pertussis test: serology; *n* (%)	69 (10.2%)	61 (10.2%)	36 (9.3%)	8 (10%)
Unknown diagnostic test for pertussis; *n* (%)	40 (5.9%)	36 (6.0%)	18 (4.6%)	4 (5%)
Result white blood cell counting; *n* (%)	225/476 (47.3%)	200/421 (47%)	141/276 (51.1%)	25/55 (45%)
White blood cell count; median (range)	17.2 (4.3–106.1)	17.7 (5.3–106.1)	17.2 (5.3–74.3)	13.5 (4.3–64.0)
Result C-reactive protein; *n* (%)	142/392 (36.2%)	119/344 (35%)	81/231 (35.1%)	23/48 (48%)
C-reactive protein; median (range)	5 (0–415)	4.7 (0–415)	5 (0–415)	15 (0–363)^c,d^
Positive co-infections^h^; *n* (%)	81/355 (22.8%)	63/306 (20.6%)	46/208 (22.1%)	18/49 (36.7%)^c,d^
Specification of prescribed antibiotics before admission				
Amoxicillin	49/132 (37%)	38/114 (33%)	23/66 (35%)	11/18 (65%)
Azithromycin	24/132 (18%)	21/114 (18%)	17/66 (26%)	3/18 (18%)
Ceftriaxone	1/132 (1%)	1/114 (1%)	1/66 (2%)	0/0
Clarithromycin	41/132 (31%)	38/114 (33%)	17/66 (26%)	3/18 (18%)
Erythromycin	7/132 (5%)	6/114 (5%)	2/66 (3%)	1/18 (6%)
Feneticillin	1/132 (1%)	1/114 (1%)	1/66 (2%)	0/0
Trimethoprim	1/132 (1%)	1/114 (1%)	1/66 (2%)	0/0
unknown	7/132 (5%)	8/114 (7%)	4/66 (6%)	0/18 (0%)
Specification of prescribed antibiotics during admission				
Amoxicillin	11/543 (2%)	10/475 (2%)	7/313 (2%)	1/68 (1%)
Azithromycin	170/543 (31%)	146/475 (31%)	98/313 (31%)	24/68 (35%)
Cephotaxim	1/543 (0.2%)	0/0	0/0	1/68 (1%)
Clarithromycin	314/543 (58%)	280/475 (59%)	184/313 (59%)	34/68 (50%)
Erythromycin	44/543 (8%)	37/475 (8%)	23/313 (7%)	7/68 (10%)
unknown	3/543 (0.6%)	2/475 (0.4%)	1/313 (0.3%)	1/68 (1%)
Specification of reported complications				
conjunctivitis	5 (0.7%)	5 (0.8%)	1 (0.3%)	0 (0%)
convulsion	5 (0.7%)	5 (0.8%)	4 (1.0%)	0 (0%)
encephalopathy	1 (0.2%)	1 (0.2%)	1 (0.3%)	0 (0%)
pneumonia	11 (1.6%)	9 (1.5%)	7 (1.8%)	2 (2.5%)
otitis media	1 (0.2%)	1 (0.2%)	0 (0%)	0 (0%)
death	2 (0.3%)	2 (0.3%)	2 (0.5%)	0 (0%)
bradycardia	8 (1.2%)	7 (1.2%)	6 (1.6%)	1 (1.3%)
cardio-respiratory insufficiency	7 (1.0%)	5 (0.8%)	4 (1.0%)	2 (2.5%)
desaturation	20 (3.0%)	18 (3.0%)	14 (3.6%)	2 (2.5%)
feeding problems	5 (0.7%)	2 (0.3%)	0 (0%)	3 (3.8%)
weight loss	12 (1.8%)	11 (1.9%)	8 (2.1%)	1 (1.3%)
dehydration	1 (0.2%)	1 (0.2%)	1 (0.3%)	0 (0%)
need for drip-feed	4 (0.6%)	2 (0.3%)	1 (0.3%)	2 (2.5%)
gastroenteritis	3 (0.4%)	2 (0.3%)	2 (0.5%)	1 (1.3%)
sepsis	1 (0.2%)	1 (0.2%)	0 (0%)	0 (0%)
extreme high white blood cell count	2 (0.3%)	1 (0.2%)	0 (0%)	1 (1.3%)
metabolic alkalosis	1 (0.2%)	1 (0.2%)	0 (0%)	0 (0%)
hypotonia	2 (0.3%)	1 (0.2%)	1 (0.3%)	1 (1.3%)

^a^term infants in main analysis

^b^term infants in sensitivity analysis

^c^significant difference (p < 0.05) between certain+probable terms and preterms (main analysis)

^d^significant difference (*p* < 0.05) between certain terms and preterms (sensitivity analysis)

^e^Specific complications are listed at the end of the table

^f^Specification of prescribed antibiotics before admission are listed at the end of the table

^g^Specification of prescribed antibiotics during admission are listed at the end of the table

^h^Specification of diagnostics for coinfections are listed in Table 2

#### Clinical course and treatment

On admittance, 93% of infants were coughing, and 73% also suffered from coughing attacks (Table 1). Other classical pertussis symptoms such as prolonged inspiratory effort, whooping, vomiting, and apneas were reported in up to 35% of infants. Reported symptoms at admission did not differ between terms and preterms, except that cyanosis more often occurred in terms (44% vs 31%; *p* = 0.03).

Complications like bradycardia, respiratory insufficiency and desaturation, feeding problems, weight loss, and pneumonia were reported in 9% of infants, with a slightly (not-significant) higher frequency in preterms (Table 1). Two term infants, too young to be eligible for vaccination, died due to pertussis.

Before admission, 20% of infants already had received antibiotics, whereas 81% received antibiotics during hospitalization (Table 1). Though preterms were slightly more often treated with intravenous antibiotics, the start and duration of antibiotics was comparable between the groups. We also found a comparable need for additional oxygen (37% vs 34%) and ICU admittance (13% vs 10%), though a trend to more often need artificial respiration was observed in preterms compared to terms (14% vs 7%; *p* = 0.05). Duration of ICU stay was longer in preterms than in terms (median 15 vs 9 days; *p* = 0.004).

At discharge, 77% of infants still had symptoms, with coughing most prevalent (Table 1). Likewise, 14% needed re-admittance within 6 weeks after discharge. Both frequencies were somewhat lower in preterms compared to terms.

#### Diagnostics

For 91% of infants, information on diagnostics to confirm pertussis was found in the medical record (Table 1). In 5% we found evidence of all three diagnostic procedures (i.e., culture, polymerase chain reaction, and serology), with two procedures in 25% and one procedure in 64% of infants.

White blood cell (WBC) counts were available for one third of the medical records; the median highest value was17.2 (range 4.3–106.1), with somewhat lower values in preterms (Table 1). Data on C-reactive protein (CRP) were available for 21% of records, with lower median CRP in terms than preterms (4.7 vs 15; *p* = 0.007).

In 53% of the infants, diagnostics were performed for pathogens other than *B. pertussis* (Tables 1 and 2). Overall, preterms were more often tested (61% vs 51%; *p* = 0.2) and diagnosed (37% vs 21%; *p* = 0.01) with coinfections than terms. Respiratory syncytial virus (RSV), influenzavirus, adenovirus, human meta-pneumovirus, para-influenzavirus, rhinovirus, and *Mycoplasma pneumoniae* were observed in 21 to 57% of tested cases, with RSV and para-influenzavirus more often observed in preterms (Table 2). In total, tests for 43 pathogens were performed. Respiratory pathogens are recorded in Table 2.

**Table 2:** Percentages (numbers tested and positive) of pathogens besides *B.pertussis* causing respiratory symptoms in term and preterm infants. Denominators are specified per cell

	Total group (*n* = 676)	Probable and certain term infants (*n* = 596)^a^	Certain term infants (*n* = 388)^b^	Preterm infants (*n* = 80)
Respiratory syncytial virus	21% (62/293)	19% (49/257)	20% (34/169)	36% (13/36)^c,d^
Influenzavirus	34% (38/112)	31% (29/93)	35% (22/62)	47% (9/19)
Adenovirus	36% (35/98)	33% (27/82)	35% (18/51)	50% (8/16)
Human metapneumovirus	35% (28/81)	32% (22/68)	32% (12/37)	46% (6/13)
parainfluenzavirus	33% (25/76))	28% (18/65)	28% (12/43)	64% (7/11)^c,d^
Rhinovirus	57% (40/70)	56% (33/59)	63% (25/40)	64% (7/11)
Mycoplasma pneumoniae	29% (19/65)	27% (15/55)	30% (10/33)	40% (4/10)
Bocavirus	56% (9/16)	42% (5/12)	44% (4/9)	100% (4/4)
*Chlamydophila pneumoniae*	41% (12/29)	48% (12/25)	60% (9/15)	0% (0/4)
*Chlamydia psittaci*	0% (0/1)	0% (0/1)	not tested	not tested
Cytomegalovirus	33% (4/12)	22% (2/9)	33% (2/6)	67% (2/3)
Coronavirus	56% (18/32)	50% (14/28)	56% (9/16)	100% (4/4)
*Coxiella burnetii*	0% (0/7)	0% (0/6)	0% (0/4)	0% (0/1)
Enterovirus	39% (9/23)	42% (8/19)	40% (6/15)	25% (1/4)
*Haemophilus influenzae*	75% (3/4)	75% (3/4)	100% (3/3)	not tested
*Klebsiella oxytoca*	100% (1/1)	not tested	not tested	100% (1/1)
*Klebsiella pneumoniae*	100% (2/2)	100% (1/1)	100% (1/1)	100% (1/1)
*Legionella*	43% (3/7)	50% (3/6)	60% (3/5)	0% (0/1)
*Moraxella catarrhalis*	100% (5/5)	100% (4/4)	100% (3/3)	100% (1/1)
*Bordetella parapertussis*	0% (0/5)	0% (0/3)	0% (0/3)	0% (0/2)
Parechovirus	0% (0/3)	0% (0/2)	0% (0/2)	0% (0/1)
*Streptococcus pneumoniae*	100% (3/3%)	100% (1/1)	100% (1/1)	100% (2/2)

^a^term infants in main analysis

^b^term infants in the sensitivity analysis

^c^significant difference between certain+probable terms and preterms (main analysis)

^d^significant difference between certain terms and preterms (sensitivity analysis)

#### Influence of coinfections and age at hospitalization on the clinical picture

Using multivariable logistic regression analysis, we studied the association between the clinical course and treatment of pertussis and prematurity. We adjusted for age at hospitalization and presence of coinfections. Results were in line with the trends mentioned in previous results sections (Table 3). With respect to the need for artificial respiration, the risk among preterms was significantly higher than in terms (adjusted Odds Ratio (OR) 2.8; 95% CI 1.3–6.0). With respect to apnea, preterms showed an increased risk after adjustment (OR 1.8; 95% CI 1.0–3.3).

**Table 3:** Multivariable logistic regression analyses of the association between premature delivery and clinical course and treatment of pertussis; crude and adjusted OR and 95% confidence intervals (95%CI)

	Probable and certain term infants (*n* = 596)^a,b^	Preterm infants (n = 80)^b^	Crude OR (95% CI)	Adjusted OR (95% CI)^c^
Symptoms at admission				
Coughing attacks	438 (73.5%)	56 (70.0%)	0.8 (0.5–1.4)	0.8 (0.5–1.4)
Apnea	92 (15.4%)	18 (22.5%)	1.6 (0.9–2.8)	**1.8 (1.0–3.3)**
Whooping	23 (3.9%)	1 (1.3%)	0.3 (0.04–2.4)	0.3 (0.04–2.3)
Vomiting	210 (35.2%)	28 (35.0%)	1.0 (0.6–1.6)	1.0 (0.6–1.6)
Prolonged inspiratory effort	42 (7.1%)	7 (8.8%)	1.3 (0.5–2.9)	1.2 (0.5–2.7)
Collapse	6 (1.0%)	2 (2.5%)	2.5 (0.5–12.7)	3.8 (0.7–19.7)
Cyanosis	259 (43.5%)	25 (31.3%)	0.6 (0.4–1.0)	0.7 (0.4–1.1)
Fever	52 (8.7%)	8 (10.0%)	1.2 (0.5–2.6)	0.9 (0.4–2.0)
Feeding problems	185 (31.0%)	27 (33.8%)	1.1 (0.7–1.9)	1.1 (0.7–1.9)
Complications				
Any complication	53 (8.9%)	10 (12.5%)	1.5 (0.7–3.0)	1.6 (0.8–3.4)
Treatment				
Antibiotics before admission	114/580 (19.7%)	18/77 (23.4%)	1.1 (0.6–2.0)	1.1 (0.6–1.9)
Antibiotics during admission	475/587 (80.9%)	68/78 (87.2%)	1.5 (0.7–3.2)	1.8 (0.9–3.9)
Artificial respiration	39/577 (6.8%)	11/77 (14.3%)	**2.3 (1.1–4.8)**	**2.8 (1.3–6.0)**
Additional oxygen	198/585 (33.9%)	28 (36.8%)	1.1 (0.7–1.9)	1.3 (0.8–2.2)
Admission intensive care unit (ICU)	59 (9.9%)	10 (12.5%)	1.3 (0.6–2.7)	1.6 (0.8–3.6)
Discharge				
Symptoms remaining	460 (77.2%)	58 (72.5%)	0.8 (0.5–1.3)	0.8 (0.5–1.4)

Term infants are set as reference. Significant results are in bold

^a^term infants in main analysis

^b^If denominator is different, it is reported in the cell

^c^adjusted for coinfections and age in months at admission

#### Influence of vaccination and vaccine effectiveness (VE)

Being vaccinated was associated with a reduction in the median duration of hospitalization among both terms (9 vs 3 days; *p* < 0.0001) and preterms (13 vs 5 days; *p* = 0.01). A lower median duration of ICU admission was found among vaccinated preterms (8 vs20 days; *p* = 0.1), but not in terms. Furthermore, the median highest WBC count was lower in vaccinated terms (*p* = 0.004), but not in vaccinated preterms. In both groups, vaccination was significantly associated with a lower crude risk of apneas and the need for artificial respiration, additional oxygen, or ICU admittance (Table 4). In term infants, vaccination appeared to reduce the crude risk of complications and prescription of antibiotics during admission, but the crude risk of antibiotic prescription before admission appeared higher among vaccinated terms. After adjustment for coinfections and age at admittance, differences were no longer significant except for the lower need of oxygen treatment in vaccinated terms.

**Table 4:** Multivariable logistic regression analysis of the association between pertussis vaccination and the clinical course and treatment of pertussis, stratified for term and preterm infants (crude and adjusted OR and 95% CI)

	Probable and certain term infants (*n* = 481)^a^				Preterm infants (*n* = 60)			
	Unvaccinated infants (*n* = 268)	Vaccinated infants (*n* = 213)	crude OR (95% CI)	adjusted OR (95% CI)^b^	Unvaccinated infants (*n* = 23)	Vaccinated infants (*n* = 37)	crude OR (95% CI)	adjusted OR (95% CI)^b^
Symptoms at admission								
Coughing attacks	197 (73.5%)	151 (70.9%)	0.9 (0.6–1.3)	0.8 (0.4–1.3)	17 (73.9%)	26 (70.3%)	0.8 (0.3–2.7)	0.8 (0.1–5.6)
Apnea	47 (17.5%)	22 (10.3%)	**0.5 (0.3–0.9)**	0.6 (0.3–1.1)	10 (43.5%)	5 (13.5%)	**0.2 (0.06–0.7)**	0.2 (0.03–1.5)
Whooping	6 (2.2%)	12 (5.6%)	2.6 (0.96–7.1)	1.8 (0.5–6.8)	1 (4.4%)	0	< 0.001 (< 0.001- > 999.9)	0.4 (< 0.001- > 999.9)
Vomiting	94 (35.1%)	79 (37.1%)	1.1 (0.8–1.6)	0.9 (0.6–1.6)	8 (34.8%)	13 (35.1%)	1.0 (0.3–3.0)	0.6 (0.1–3.3)
prolonged inspiratory effort	15 (5.6%)	19 (8.9%)	1.7 (0.8–3.3)	1.0 (0.4–2.3)	1 (4.4%)	4 (10.8%)	2.7 (0.3–25.4)	1.6 (0.1–26.1)
Collapse	2 (0.8%)	2 (0.9%)	1.3 (0.2–9.0)	7.2 (0.2–226.7)	1 (4.4%)	1 (2.7%)	0.6 (0.04–10.3)	0.6 (< 0.001- > 999.9)
Cyanosis	126 (47.0%)	88 (41.3%)	0.8 (0.6–1.1)^c^	1.1 (0.7–1.8)^c^	5 (21.7%)	17 (45.9%)	3.1 (0.9–10.0)^c^	1.9 (0.3–11.6)^c^
Fever	17 (6.3%)	21 (9.9%)	1.6 (0.8–3.1)	0.7 (0.3–1.7)	2 (8.7%)	4 (10.8%)	1.3 (0.2–7.6)	> 999.9 (< 0.001- > 999.9)
Feeding problems	87 (32.5%)	63 (29.6%)	0.9 (0.6–1.3)	0.9 (0.5–1.4)	10 (43.5%)	12 (32.4%)	0.6 (0.2–1.8)	0.8 (0.2–4.5)
Complications								
Any complication	30 (11.2%)	9 (4.2%)	**0.4 (0.2–0.8)**	0.9 (0.4–2.5)	5 (21.7%)	3 (8.1%)	0.3 (0.07–1.5)	0.2 (0.01–4.6)
Treatment								
Antibiotics before admission	37 (14.2%)	52 (25.1%)	**1.8 (1.2–2.8)**	1.2 (0.7–2.1)	2 (9.5%)	9 (24.3%)	2.7 (0.6–11.4)	12.3 (0.8–188.3)
Antibiotics during admission	230 (87.5%)	157 (74.4%)	**0.4 (0.3–0.7)**	0.7 (0.4–1.3)	21 (95.5%)	32 (86.5%)	0.3 (0.03–2.8)	1.7 (0.1–23.7)
Artificial respiration	23 (8.9%)	5 (2.4%)	**0.3 (0.1–0.7)**	0.8 (0.2–3.4)	7 (31.8%)	2 (5.6%)	**0.1 (0.02–0.7)**	0.4 (0.02–5.7)
Additional oxygen	133 (50.4%)	33 (15.9%)	**0.2 (0.1–0.3)**	**0.3 (0.2–0.5)**	13 (59.1%)	10 (27.8%)	**0.2 (0.08–0.8)**	0.6 (0.09–4.0)
Admission intensive care unit (ICU)	42 (15.7%)	5 (2.4%)	**0.1 (0.05–0.3)**	0.3 (0.1–1.1)	6 (26.1%)	2 (5.4%)	**0.2 (0.03–0.9)**	0.6 (0.04–7.6)
Discharge								
Symptoms remaining	206 (76.9%)	162 (76.1%)	0.9 (0.6–1.3)	0.7 (0.5–1.2)	14 (60.9%)	30 (81.1%	1.9 (0.7–5.1)	2.1 (0.7–6.7)

Unvaccinated infants are set as reference. Significant results are in bold

^a^term infants in main analysis

^b^adjusted for coinfections and age in months at admission

^c^crude and adjusted OR for cyanosis in term and preterms are significantly different (*p* = 0.03 and *p* = 0.02, respectively)

Among preterms, VE of the first infant dose (i.e., at 2 months of age) was 73% (95%CI 20–91%) compared to 95% (95% CI 93–96%) among terms (Table 5). Effectiveness of the second dose of the primary vaccination series was comparable in both groups at 86 and 99%, respectively.

**Table 5:** Number of infants vaccinated at admission, monthly cumulative coverage estimates, and VE against pertussis hospitalizations of 1st and 2nd infant dose for preterm and term infants, assessed with the screening method

	Term infants			Preterm infants		
Age in months↓	Vaccinated at admission % (*n*)	Coverage in general population	Vaccine effectiveness (95% CI)^a^	Vaccinated at admission % (*n*)	Coverage in general population	Vaccine effectiveness (95% CI)^a^
Main analysis						
0 m	0% (0/1)	na	na	0% (0/1)	na	na
1 m	1.4% (2/144)	1.9%	na	0% (0/14)	1.3%	na
2 m	34.2% (52/152)	90.9%	95%^b^ (93–96%)	60% (9/15)	84.9%	73%^b^ (20–91%)
3 m	85.9% (67/78)	98.8%	93% (85–96%)	86.7% (13/15)	97.9%	86% (9–96%)
4 m	93.8% (30/32)	99.4%	91%	100% (4/4)	99.4%	na
Sensitivity analysis						
0 m	0% (0/1)	na	na	0% (0/1)	na	na
1 m	0.9% (1/115)	1.9%	na	0% (0/14)	1.3%	na
2 m	33.7% (33/98)	90.9%	95%^b^ (92–97%)	60% (9/15)	84.9%	73%^b^ (20–91%)
3 m	89.4% (42/47)	98.8%	90% (71–96%)	86.7% (13/15)	97.9%	86% (9–96%)
4 m	100% (14/14)	99.4%	na	100% (4/4)	99.4%	na

^a^according to the screening method

^b^significant difference between term and preterm infants

### Sensitivity analyses; n = 468

Taking into account only those infants with known GA, findings were comparable to the main analyses, i.e., analyses in relation to prematurity (*n* = 468), vaccination status (*n* = 379), and VE estimates (not all data shown) (Tables 1, 2 and 5).

## Discussion

This medical record study on infant pertussis hospitalizations showed an overrepresentation of preterms, who accounted for 12% of all pertussis hospitalizations while comprising, on average, 8% of Dutch birth cohorts [21]. Furthermore, preterms were older than terms at hospitalization and had more often received their first pertussis vaccinations. Effectiveness of this first dose was significantly lower for preterms than for terms. Moreover, preterms tended to need more often intensive treatment and had a longer ICU stay. Likewise, preterms tended to be diagnosed more often with coinfections. Despite lower VE, the first vaccination against pertussis reduced disease severity and the need for intensive treatment in both groups. Coinfections and age at admission influenced the need for intensive treatment and mitigated the beneficial effect of pertussis vaccination.

The overrepresentation of preterms has been reported in other studies. In Norway, 10% of infant pertussis hospitalizations concerned preterms, compared to 5% born prematurely nationwide [22]. Similar data are derived from England [13], Australia [23], and Canada [24]. Low birth weight (LBW), which is associated with preterm delivery, was increased among hospitalized pertussis cases in Jerusalem [25]. Likewise, Langkamp et al. showed that LBW infants were at increased risk of pertussis hospitalizations compared to those of normal birth weight [26]. Winter et al. reported that LBW and low GA were associated with increased risk of fatal pertussis [4].

The clinical picture in our observational study resembles findings in other retrospective studies. Marshall et al. showed that preterms had a higher pertussis disease severity score (defined by a longer hospital stay, ICU admittance, need for rehydration, respiratory support, coinfections, and the presence of complications) than terms [23]. In England, a longer duration of hospitalization and higher frequencies of ICU admittance and coinfections were observed among preterms, although their frequency of coinfections was lower than we found (10% vs 37%) [13].Langkamp et al. reported a higher median age at hospitalization and a longer median length of stay among LBW infants than among those of normal birth weight [26].

Compared to our study, more active and prospective study designs have revealed higher frequencies of clinical characteristics in infants hospitalized for pertussis (both term and preterm) like cyanosis/desaturation (72%) and apnea (33%) at admittance [25]. Australian researchers observed higher rates of ICU admittance (18%) and treatment with antibiotics (96%) but lower median length of ICU stay (6 days) [27]. In Switzerland, hospitalized infants < 6 m of age had higher frequencies of coughing attacks (93%), whooping (69%), vomiting (59%) and complications (24%) [28, 29]. As active designs usually profit from more structured clinical observations and documentation in the medical record, the frequencies found in retrospective studies probably underestimate the pertussis burden in terms and preterms [30].

The aim of vaccination against pertussis is to prevent severe disease. Our study confirms that vaccination reduced disease severity and duration of hospitalization, in line with findings in other countries [27, 31–33] and findings in the Netherlands in 2006–2008 [6]. However, VE was found to be higher in our study than, for example, studies in Germany (VE 68%; 95%CI 46–81) and New Zealand (VE 43%; 95%CI 21–58) report [34, 35]. Unfortunately, no data on GA were provided in those studies. In Scandinavia, the VE of the first pertussis vaccination against hospitalization did not differ between terms and preterms, being 61% vs 71% in Norway and 51% vs 45% in Denmark [22, 36]. Both countries start their immunization schedule at age 3 months as compared to the Netherlands, where it occurs between 6 weeks to 2 months.

In 2-month-olds, protective maternal antibodies may be higher than in 3-month-olds, and higher in terms than in preterms [37]. Likewise, studies have shown a decreased immune response after immunization at 2 months in preterms compared with terms [38, 39] while finding an equal response at 3 months [40]. The fact that the Scandinavian countries saw no difference in VE between term and preterms might be explained by the interplay between maternal antibodies that reduce a first response and an immune response that is ineffective in preterms at age 2 months but may have improved by age 3 months [41]. For the 2nd dose, we found no VE difference between terms and preterms.

As in other studies, we found that the effect of vaccination on the clinical course was influenced by coinfections [13, 23], which included viral infections like RSV [42–47]. However, a recent systematic review concluded that the influence of coinfections on pertussis disease severity remains unclear [48].

Our study has several strengths. It used nationwide data collected over a 10-year period. The data contained detailed information on clinical characteristics of pertussis, also including information on possible confounding factors, e.g., coinfections. Furthermore, linkage to the vaccination registry enabled us to use validated vaccination records. Finally, the vast majority of the included pertussis cases were laboratory confirmed.

Limitations of our study include the retrospective design and the institutional differences in pertussis diagnostics, diagnostics for coinfections, and registration of clinical and laboratory disease characteristics. The 50% participation rate of hospitals might have influenced our results, but participating hospitals were spread over the country and showed a good representation of tertiary, top clinical, and local hospitals in the Netherlands. Results might have been influenced by the incomplete reporting of hospital diagnoses at discharge, assessed in a recent capture-recapture analysis [8]. We could not stratify this underreporting by GA, but the good representation of tertiary, top clinical, and local hospitals in our study probably led to inclusion of a representative variety in the spectrum of pertussis disease. This conclusion is underlined by our finding that preterms are overrepresented, as found by other studies.

Further limitations include the retrospective use of medical records, which led to missing data, e.g., on GA, birth weight, with possible impact on our results. For this reason our main analysis was based on all records, assuming that infants with unknown GA were born term. Our sensitivity analysis showed no major impact of missing data on GA, thereby confirming our findings. A breakdown of preterms into smaller groups based on GA would be clinically relevant, but low numbers prohibited this analysis. Likewise, WBCs and CRP data were incomplete, also leading to less informative results.

Linkage between medical records and vaccination data, based on pseudonymization, might have led to error. Medical records include the home address at the patient’s last visit, whereas the vaccination registry includes the current home address. Especially in cases of frequent moving and/or a large interval since the last visit, linkage might be incorrect. In our study, 38% of infants did not move house. Furthermore, the use of additional pseudonyms based on previous home addresses (also stored in the vaccination registry) lowered the risk of incorrect linking.

## Conclusion

Preterms were overrepresented among pertussis hospitalizations and had a slightly higher overall risk of complications, increased need for intensive treatment, and lower effectiveness of the first infant pertussis vaccination. The Dutch Health Council recommended maternal pertussis vaccination in the 3rd trimester of pregnancy. While this strategy is overall very effective, preterms may benefit less due to less protective maternal antibody transfer before delivery [12, 13]. They probably will benefit more from 2nd trimester immunization. Especially for infants born of unvaccinated mothers, a timely first post-natal dose remains important, as it greatly reduces cases of pertussis and decreases disease severity. Our study underlines the need for more in-depth surveillance of vaccine-preventable diseases in relation to GA and more insight into optimizing the vaccination program for all children but in particular for preterms, the most vulnerable group.

## Data Availability

The datasets generated and/or analyzed during the current study are not publicly available due to privacy legislation. A fully anonymized dataset of the 676 infants is available from the corresponding author on reasonable request. Depending on the research question, the dataset will be condensed as much as possible.

## Abbreviations

CRP: C-reactive protein
GA: Gestational age
ICD: International Classification of Diseases
ICU: Intensive care unit
LBW: Low birth weight
OR: Odds Ratio
RSV: Respiratory syncytial virus
VE: Vaccine effectiveness
WBC: White blood cell
